# Dual-slope imaging of cerebral hemodynamics with frequency-domain near-infrared spectroscopy

**DOI:** 10.1117/1.NPh.10.1.013508

**Published:** 2023-01-02

**Authors:** Giles Blaney, Cristianne Fernandez, Angelo Sassaroli, Sergio Fantini

**Affiliations:** Tufts University, Department of Biomedical Engineering, Medford, Massachusetts, United States

**Keywords:** functional near-infrared spectroscopy, diffuse optical imaging, dual-slope, frequency-domain near-infrared spectroscopy, coherent hemodynamics spectroscopy, brain hemodynamics

## Abstract

**Significance:**

This work targets the contamination of optical signals by superficial hemodynamics, which is one of the chief hurdles in non-invasive optical measurements of the human brain.

**Aim:**

To identify optimal source–detector distances for dual-slope (DS) measurements in frequency-domain (FD) near-infrared spectroscopy (NIRS) and demonstrate preferential sensitivity of DS imaging to deeper tissue (brain) versus superficial tissue (scalp).

**Approach:**

Theoretical studies (*in-silico*) based on diffusion theory in two-layered and in homogeneous scattering media. *In-vivo* demonstrations of DS imaging of the human brain during visual stimulation and during systemic blood pressure oscillations.

**Results:**

The mean distance (between the two source–detector distances needed for DS) is the key factor for depth sensitivity. *In-vivo* imaging of the human *occipital* lobe with FD NIRS and a mean distance of 31 mm indicated: (1) greater hemodynamic response to visual stimulation from FD phase versus intensity, and from DS versus single-distance (SD); (2) hemodynamics from FD phase and DS mainly driven by blood flow, and hemodynamics from SD intensity mainly driven by blood volume.

**Conclusions:**

DS imaging with FD NIRS may suppress confounding contributions from superficial hemodynamics without relying on data at short source–detector distances. This capability can have significant implications for non-invasive optical measurements of the human brain.

## 1 Introduction

Functional brain diffuse optical imaging (DOI) using near-infrared spectroscopy (NIRS) has seen an increase in its popularity and applications over the past 30 years.^1^^–^^3^ During that time, functional near-infrared spectroscopy (fNIRS) has been demonstrated in both behavioural and social studies^2^ and in clinical applications.^3^ A large reason for the success of fNIRS is due to its ability to spatially map brain hemodynamics and activation in specific cortical regions while being non-invasive, portable, and low-cost especially when comparing the latter two advantages to functional magnetic resonance imaging (fMRI).^4^ Moreover, NIRS offers continuous monitoring of key target organs, not only at the bedside but in real-life settings.^3^ However, fNIRS and DOI still struggle with one of their largest weaknesses, a significant sensitivity to superficial, extracerebral tissue.^5^^–^^8^ Despite the aforementioned advantages of DOI, most techniques still preferentially measure scalp and skull hemodynamics, with only a weak contribution from the brain itself. Therefore, the field has continually investigated methods that seek to identify or suppress this superficial signal and allow for more specific brain measurements.^7^^–^^12^

The cheapest and most common implementations of fNIRS and DOI have utilized continuous-wave (CW), methods that are most strongly affected by superficial hemodynamics. In frequency-domain (FD)^13^ or time-domain (TD)^14^ techniques, the phase (ϕ) or higher moments of the photon time-of-flight distribution, respectively, intrinsically provide measurements that are more specific to deep regions. Despite this, due to the widespread use of fNIRS, a majority of the aforementioned techniques to determine the brain’s contribution to the signal are targeted toward CW data and include measurements that are specifically sensitive to superficial hemodynamics.^7^^–^^10^ Recently, there has been a push to implement imaging arrays using FD or TD NIRS to gain ϕ or higher moments information in an attempt to retrieve optical data that are intrinsically sensitive to deeper tissue.^11^^,^^12^^,^^15^^–^^17^ Furthermore, typical implementations of DOI utilize single-distance (SD) based source–detector arrangements that consist of source–detector pairs spaced at various source–detector distances (ρs) across the SD sets.^4^^,^^18^ SD measurements are known to be largely sensitive to superficial tissue. To combat this problem, the combination of data collected at different ρs or many SDs has been used to minimize signal contributions associated with superficial tissue in some way.^4^^,^^7^^–^^10^^,^^12^^,^^16^^,^^17^ However, it is still unclear which set of ρs will optimally reconstruct deep tissue dynamics. A method that has been introduced to achieve this subtraction intrinsically is the dual-slope (DS).^11^^,^^19^ One of the main differences between this technique and others, is its use of only relatively long ρs (≥25  mm) with the hypothesis that data collected at different long ρs will feature comparable contributions from superficial (scalp) tissue and different contributions from deeper (brain) tissue. This DS technique has been applied primarily to FD data^11^^,^^13^^,^^19^ and also has been proposed in TD.^15^

The typical DS configuration consists of two sources and two detectors, which realize symmetric measurements of two slopes of optical data versus ρ.^20^^,^^21^ These slopes are averaged to achieve DS measurements that feature a Sensitivity to absorption change (S) selective to deeper tissue,^11^^,^^19^^,^^20^ and also supress artifacts from changes in the probe-tissue coupling or from instrumental drifts [inherited from the self-calibrating (SC) method].^22^^,^^23^ In FD fNIRS, one measures a complex reflectance ($\overset{˜}{R}$) corresponding to the modulation frequency ($f_{\mathrm{mod}}$) of the source. The slopes of optical data used in FD DSs are proportional to the differences between measurements at different ρs of either the linearized complex reflectance amplitude ($\ln(\rho^{2}|\overset{˜}{R}|)$) (also referred to as linearized intensity (I) since $\left|\overset{˜}{R}\right|$ is equivalent to I) or the phase of the complex reflectance ($∠\overset{˜}{R}$) (referred to as ϕ). DS also inherits the ability of the SC technique to preform calibration-free measurements of absolute optical properties of tissue, namely the absorption coefficient ($\mu_{a}$) and the reduced scattering coefficient ($\mu_{s}^{\prime }$), when the dual slopes of I and ϕ are used in combination.^22^

This work seeks to apply DS methods to DOI *in-vivo*, bringing with it all of the expected advantages of DS. Prior to this work, DS DOI had been applied to optical phantoms, showing that DS ϕ is able to preferentially reconstruct deep perturbations even in the presence of a superficial perturbation.^24^ Extensive work was then done to develop methods to design DS DOI arrays,^21^ resulting in the construction of a DS array for large area coverage in fNIRS DOI. The methods used in Ref. 21 did not include an analysis on the effect of ρs on the Sensitivity to absorption change (S) (i.e., the ratio between a measured absorption coefficient change($\Delta \mu_{a}$) and a true $\Delta \mu_{a}$ localized within the medium) to top- and bottom- layers, but instead focused on meeting practical requirements based on instrumental limits. Herein, the novel aspects are the determination of optimal source–detector distances and first applications of a DS DOI array for DS mapping of cerebral hemodynamics *in-vivo*. The results presented here allow for the investigation of *in-vivo* spatial maps of DS I and ϕ, as compared to previously reported single-location DS measurements,^11^^,^^25^ and show the applicability of this novel DS array to imaging the human brain.

In this paper, three experiments investigating DS for DOI are presented. First, an *in-silico*, theoretical investigation of the ρs in a DS set using an analytical solution to the diffusion equation for two-layer media.^26^^,^^27^ This experiment is an extension of the work in Ref. 21 with the goal to further examine the choices made in the DS array design, and a special emphasis on the optimal ρs for DS measurements. The second and third experiments are the first *in-vivo* demonstrations of DS DOI on the human brain. The second experiment is a standard visual stimulation protocol^28^ whose primary aim is to compare the functional hemodynamic response recorded in the primary visual cortex using different DS and SD fNIRS data-types. The third experiment seeks to demonstrate DS DOI of the human brain during systemic arterial blood pressure (ABP) oscillations in a standard coherent hemodynamics spectroscopy (CHS) protocol.^11^^,^^29^ This third experiment is the first CHS imaging application to be presented. It is noted that the emphasis of this work is on technology development and the demonstration of the novel DS DOI technique for mapping hemodynamics in the human brain. Therefore, a single subject was investigated, and more detailed studies of the temporal and spatial features of the cerebral hemodynamics measured with DS DOI are left to future research conducted on multiple subjects.

## 2 Methods

### 2.1 Experiments

#### 2.1.1 In-silico simulations of two-layer & homogeneous media

The first part of this work investigates how the ρs used in FD NIRS measurements affect the depth of the Sensitivity to absorption change (S). The focus is on DS^11^^,^^19^ measurements, which utilize a set of at least two ρs.^20^^,^^21^ Therefore an examination of how the maximum or mean ρ (i.e., $\rho_{\max}$ or $\overset{¯}{\rho }$, respectively) in a DS set affect the depth distribution of S was done. To this aim, two sets of diffusion theory based *in-silico* simulations for various ρs in a linear-symmetric DS set.^20^^,^^21^ For the first set, $\overset{¯}{\rho }$ was held constant at 30 mm and the difference between the ρs (i.e., Δρ) was varied from 5 to 50 mm (Table 1(left)). In the second set of simulations, $\rho_{\max}$ was held constant at 35 mm and Δρ was varied from 1 to 28 mm [Table 1(right)].

**Table 1:** Source–detector distances in the *in-silico* simulations.

Fix $\overset{¯}{\rho }=30\;\mathrm{mm}$			Fix $\rho_{\max}=35\;\mathrm{mm}$		
ρs (mm)	$\bar{\rho }$ (mm)	Δρ (mm)	ρs (mm)	$\overset{¯}{\rho }$ (mm)	Δρ (mm)
[27.5, 32.5]	30.0	5.0	[7.0, 35.0]	21.0	28.0
[25.0, 35.0]	30.0	10.0	[10.0, 35.0]	22.5	25.0
[22.5, 37.5]	30.0	15.0	[13.0, 35.0]	24.0	22.0
[20.0, 40.0]	30.0	20.0	[16.0, 35.0]	25.5	19.0
[17.5, 42.5]	30.0	25.0	[19.0, 35.0]	27.0	16.0
[15.0, 45.0]	30.0	30.0	[22.0, 35.0]	28.5	13.0
[12.5, 47.5]	30.0	35.0	[25.0, 35.0]	30.0	10.0
[10.0, 50.0]	30.0	40.0	[28.0, 35.0]	31.5	7.0
[7.5, 52.5]	30.0	45.0	[31.0, 35.0]	33.0	4.0
[5.0, 55.0]	30.0	50.0	[34.0, 35.0]	34.5	1.0

Source–detector distances (ρs), mean ρ ($\overset{¯}{\rho }$), max ρ ($\rho_{\max}$), and difference between ρs (Δρ).

For each set of ρs, more than sixteen thousand (16,807) analytical two-layer simulations were conducted with differing top-layer thickness ($z_{\text{top}}$) and absolute optical properties (i.e., $\mu_{a}$ and $\mu_{s}^{\prime }$ of each layer).^26^^,^^27^ To achieve this, the five two-layer parameters were varied through seven values and all combinations simulated. The $\mu_{a}s$ of each layer were varied in the range 0.005 to $0.015\;{\mathrm{mm}}^{-1}$, the $\mu_{s}^{\prime }s$ in the range 0.5 to $1.5\;{\mathrm{mm}}^{-1}$, and $z_{\text{top}}$ in the range 5 to 15 mm. A representative semi-infinite homogeneous medium was also simulated.^11^^,^^30^ This medium had a $\mu_{a}$ of $0.010\;{\mathrm{mm}}^{-1}$ and a $\mu_{s}^{\prime }$ of $1\;{\mathrm{mm}}^{-1}$.

Every simulation considered an $\Delta \mu_{a}$ of $0.001\;{\mathrm{mm}}^{-1}$ in the bottom-layer and a $\Delta \mu_{a}$ of $-0.001\;{\mathrm{mm}}^{-1}$ in the top-layer. Then, the methods discussed below in Secs. 2.2.1 and 2.2.2 were used to simulate the measured $\Delta \mu_{a}$ (i.e., the effective $\Delta \mu_{\mathrm{ameas}}$ obtained from the data assuming that the medium is semi-infinite and homogeneous) considering either the DS data or the long SD data, for both I and ϕ. Since preferentially deep S is desired for non-invasive brain measurements, a recovered $\Delta \mu_{\mathrm{ameas}}$ was considered better when it was closer to $0.001\;{\mathrm{mm}}^{-1}$ (the actual bottom layer $\Delta \mu_{a}$). For the representative homogeneous medium, the S was calculated using diffusion theory according to the methods described in Ref. 11 and $\Delta \mu_{\mathrm{ameas}}$ was considered as the average over all possible $z_{\text{top}}$ dividing the top- and bottom-layer perturbations. Comparison to a representative homogeneous medium was done to connect these results to previous work and conclusions drawn from the homogeneous case.^20^ Additionally, it should be noted that the homogeneous case is still relevant to this work since a homogeneous model is at the core of the DS recovery of $\Delta \mu_{\mathrm{ameas}}$ (Secs. 2.2.1 and 2.2.2) and the DS DOI methods (Sec. 2.2.4). The results from this *in-silico* experiment are shown in Sec. 3.1.

#### 2.1.2 In-vivo brain measurements

##### Equipment and human subject

All *in-vivo* measurements were performed using an FD NIRS ISS Imagent V2 (Champaign, Illinois) (Imagent), which utilizes two optical wavelengths (λs) (830 nm and 690 nm) and a $f_{\mathrm{mod}}$ of 140.625 MHz. For this work, the Imagent was configured to use 16 source pairs (two λs, thus 32 laser diodes total) and 10 detectors with a collection sampling rate of 4.96 Hz.

One healthy human subject (28 year old male) was recruited for two Tufts University institutional review board approved protocols (expounded upon below). The first protocol consisted of visual stimulation (experiment repeated three times) and the second involved systemic ABP oscillations (experiment repeated four times). The data presented here are representative of the repeated experiments, which generated similar results. It is noted that only one subject was chosen for this work since the goal is not to draw conclusions about the specific spatio-temporal characteristics of the functional or physiological cerebral hemodynamics measured, but rather to demonstrate the design, advantages, and applicability to the human brain of a DS array.

**Fig. 1:**
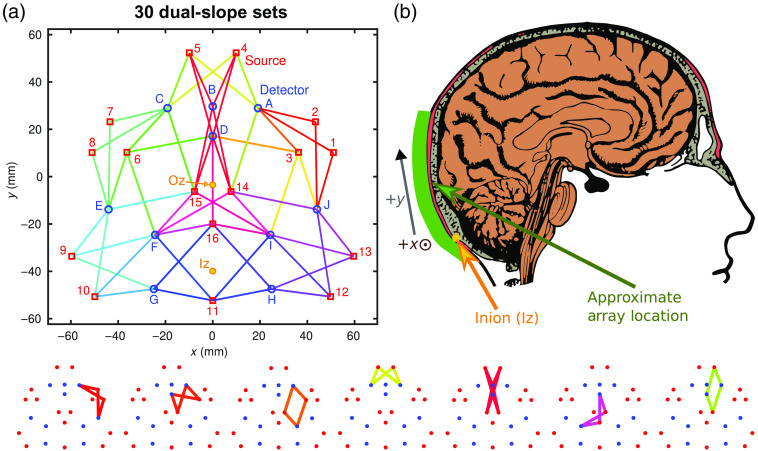
(a) Schematic of DS array where lines of differing colors show different DS sets. Examples of individual DS sets are shown along the bottom edge of the figure. The approximate Iz and Oz locations are shown in yellow. The subject’s Iz to Nz distance was 365 mm. A full list of DS sets can be found in Fig. 2. (b) Placement of the DS array during the *in-vivo* brain measurements showing the upper portion of the array primarily probing the occipital lobe.

For both protocols, the DS array described in Ref. 21 was placed on the back of the subject’s head so that the upper part of the array was over the occipital lobe [Fig. 1(b)]. In each experimental repetition, the optical array was placed in approximately the same region as shown in Fig. 1(b). The subject’s inion (Iz) to nasion (Nz) distance was ∼365  mm, and the array locations corresponding to the Iz and occipital zero (Oz) are shown in Fig. 1(a). This array consisted of 57 SD sets and 30 DS sets [Fig. 1(a)]. The array had an overall triangular shape, and covers an area of ∼120  mm on a side (about $7200\;{\mathrm{mm}}^{2}$). All of the DS ρs pairs were ∼25 and 37 mm since this array was designed to homogenize ρs.^21^ This design choice is based on the simulations described in Secs. 2.1.1 and 3.1 in this work.

**Fig. 2:**
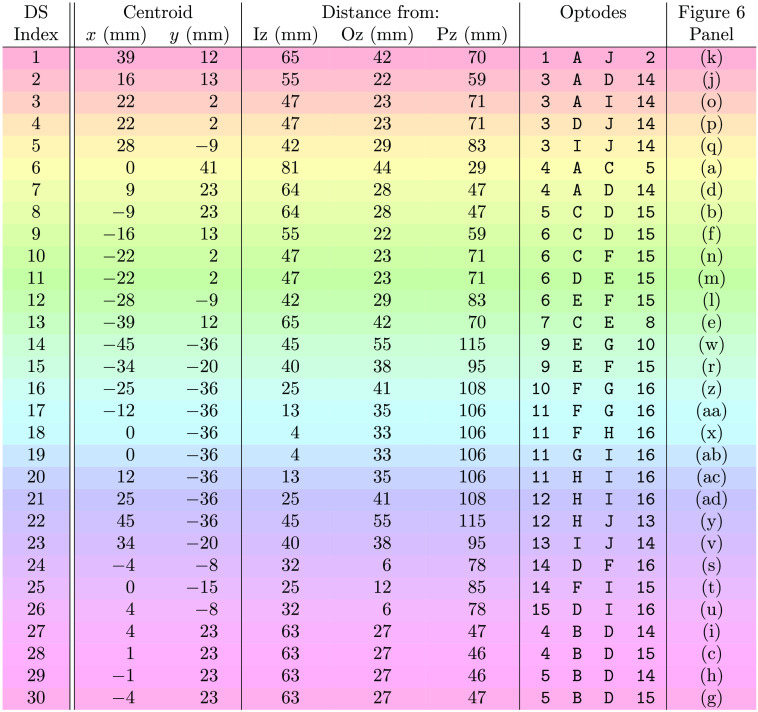
Key of dual-slope set indexes, locations, and optodes (Fig. 1). Note 1: row colors correspond to the DS set colors in Figs. 1 and 6–8. Note 2: optodes refer to ones labeled in Fig. 1, numbers are sources and letters are detectors. Acronyms: DS, Dual-slope; Iz, inion; Oz, occipital zero; and Pz, parietal zero.

##### Visual stimulation

The first *in-vivo* experiment consisted of a visual stimulation protocol. This protocol included an initial baseline and a final recovery baseline, 1 min each, from which absolute optical properties (i.e., $\mu_{a}$ and $\mu_{s}^{\prime }$) were obtained (Sec. 2.2.1). The functional activation portion of the protocol consisted of 11 stimulation-rest blocks, where the stimulation lasted 15 s and rest lasted 30 s [Fig. 1(a)]. Visual stimulation consisted of a contrast reversing circular checkerboard (ø0.65 m), which reversed at a frequecy of 8 Hz^28^ and was presented in front of the subject at a distance of 1.8 m. Results from this protocol are found in Sec. 3.2.

##### Systemic blood pressure oscillations

**Fig. 3:**
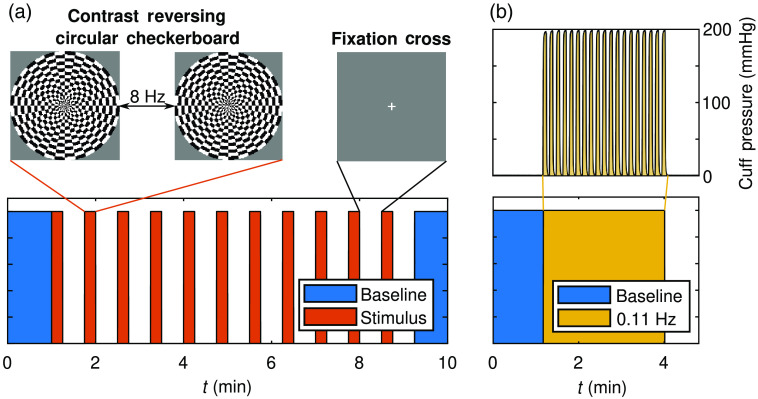
(a) Visual stimulus protocol with 11 stimulus periods (15 s stimulus preceding 30 s rest) using a contrast reversing circular checkerboard, reversing at 8 Hz. (b) Systemic blood pressure oscillations protocol where oscillations at 0.11 Hz lasted for 3 min. The pneumatic thigh cuff was set to 200-mm Hg during the cyclic occlusions.

The second *in-vivo* experiment consisted of a systemic ABP oscillation protocol.^11^ Systemic ABP oscillations were induced at a frequency (f) of 0.11 Hz using a Hokanson CC17 (Bellevue, Washington) (cuff) secured on the upper portion of each of the subject’s thighs. The dimensions of each cuff were $180\times 1080\;{\mathrm{mm}}^{2}$ when laid flat. The cuffs were placed so that they were centered on the thighs and secured not to shift during the inflation and deflation procedures. The amplitude of the cuff pressure oscillations was set to 200-mm Hg [Fig. 3(b)]. Continuous ABP measurements were taken throughout the experiment using a CNSystems CNAP 500 (Graz, Austria) (CNAP). The CNAP achieved these ABP measurements using finger plethysmography. This experimental protocol started with a 1 min baseline that was used to find baseline tissue optical properties (i.e., $\mu_{a}$ and $\mu_{s}^{\prime }$; Sec. 2.2.1). Following the initial baseline, the oscillation sequence began and lasted 3 min, leading to 19 oscillation periods at the set frequency of 0.11 Hz. The results from this experiment are presented in Sec. 3.3.

### 2.2 Analysis

#### 2.2.1 Recovery of absolute optical properties

Tissue absolute $\mu_{a}$ and $\mu_{s}^{\prime }$ were calculated for each DS set, both for the *in-silico* simulations and throughout the DS array *in-vivo* measurements. This was achieved using the DS set in SC FD-NIRS mode. To convert the FD slopes to $\mu_{a}$ and $\mu_{s}^{\prime }$, an iterative method^31^ based on a semi-infinite homogeneous medium and extrapolated boundary conditions^30^ was used. Briefly, this method uses the $\overset{˜}{R}$ versus ρ, an initial guess of the complex effective attenuation coefficient (${\overset{˜}{\mu }}_{\mathrm{eff}}$) using assumptions of linearity,^32^ and finds $\mu_{a}$ and $\mu_{s}^{\prime }$ by iteratively solving the analytical equation for $\overset{˜}{R}$ in a semi-infinite homogeneous medium.^31^ The iteratively recovered ${\overset{˜}{\mu }}_{\mathrm{eff}}$ was then converted to $\mu_{a}$ and $\mu_{s}^{\prime }$ for each DS set.

#### 2.2.2 Measuring changes in hemoglobin concentration

Dynamic changes in I or ϕ for SD or DS were translated into $\Delta \mu_{a}$ using methods reported in Ref. 11. For SD, $\Delta \mu_{a}$ was calculated using the differential path-length factor (DPF) obtained using the absolute $\mu_{a}$ and $\mu_{s}^{\prime }$ calculated as described in Sec. 2.2.1. In the case of DS, $\Delta \mu_{a}$ was calculated using the differential slope factor (DSF) obtained from said $\mu_{a}$ and $\mu_{s}^{\prime }$. These measured $\Delta \mu_{a}s$ at two λs were converted to oxy-hemoglobin concentration change (ΔO) and deoxy-hemoglobin concentration change (ΔD) using known hemoglobin extinction coefficients and Beer’s law.^33^

#### 2.2.3 Phasor analysis

The systemic ABP oscillations experiment described in Sec. 3.3 required transfer function analysis for interpretation. This was performed for each SD and DS set and each data-type (I or ϕ) independently. The analysis was done to retrieve a phasor ratio vector between oxy-hemoglobin and ABP ($\overset{˜}{O}/\overset{˜}{A}$), and a phasor ratio vector between deoxy-hemoglobin and ABP ($\overset{˜}{D}/\overset{˜}{A}$) at the induced frequency of 0.11 Hz. These vectors represented the amplitude ratio (i.e., modulus) and the phase difference (i.e., argument) of the two signals considered. To achieve this, the continuous wavelet transform (CWT) [cwt function in MathWorks MATrix LABoratory (Natick, Massachusetts) (MATLAB)], based on a complex Morlet mother wavelet, was taken of the temporal [i.e., time (t)] signals ΔO, ΔD, and ABP change (ΔA). The wavelet coefficients were interpreted as phasor maps of the Oxy-hemoglobin phasor ($\overset{˜}{O}$), Deoxy-hemoglobin phasor ($\overset{˜}{D}$), and ABP phasor ($\overset{˜}{A}$) over t and f. Then the quotient from division of the corresponding phasor maps created the transfer functions $\overset{˜}{O}/\overset{˜}{A}$ and $\overset{˜}{D}/\overset{˜}{A}$ also over t and f.

To identify which t and f regions to use in further analysis, the wavelet Coherence between oxy-hemoglobin and ABP phasors ($C(\overset{˜}{O},\overset{˜}{A})$) and the coherence between deoxy-hemoglobin and ABP phasors ($C(\overset{˜}{D},\overset{˜}{A})$) were calculated using a modified version of the MATLAB wcoherence function, which removes smoothing in f. A coherence (C) threshold generated from the 95th percentile (i.e., α=0.05) of C between random surrogate data^34^ was used to mask both $C(\overset{˜}{O},\overset{˜}{A})$ and $C(\overset{˜}{D},\overset{˜}{A})$ maps so that only ts and fs with significant coherence between the considered signals contained Boolean true. Next, a logical AND was taken between both threshold-ed $C(\overset{˜}{O},\overset{˜}{A})$ and $C(\overset{˜}{D},\overset{˜}{A})$ Boolean maps, so that only ts and fs in which both ΔO and ΔD were coherent with ΔA retained true Boolean values.^35^^,^^36^ This Boolean map of significant C was then used to mask the $\overset{˜}{O}/\overset{˜}{A}$ and $\overset{˜}{D}/\overset{˜}{A}$ transfer function maps, allowing only transfer function relationships of significant coherence to be considered in the analysis.

To select only fs around the induced frequency of 0.11 Hz, the bandwidth of a test sinusoid extending the duration of the protocol was found to be 0.02 Hz using the full-width half-max of the CWT amplitude.^36^ Finally, the significant C masked transfer functions, $\overset{˜}{O}/\overset{˜}{A}$ and $\overset{˜}{D}/\overset{˜}{A}$, were averaged within this f band and during the induced oscillation t window [Fig. 3(b)]. Therefore, the results reflected measured hemodynamics that featured significant C for both $C(\overset{˜}{O},\overset{˜}{A})$ and $C(\overset{˜}{D},\overset{˜}{A})$ at the frequency induced (0.11 Hz).

#### 2.2.4 Image reconstruction

##### General imaging methods

For image reconstruction the Moore–Penrose inverse (MP) was implemented with Tikhonov regularization (scaling parameter a=1).^21^^,^^24^ Reconstruction was conducted on the SD I, SD ϕ, DS I, and DS ϕ data separately, creating a different image for each data-type and allowing comparisons between them. 57 SD and 30 DS sets existed in the array (Fig. 1); however only sets that passed data quality requirements were considered for reconstruction (Sec. 2.2.5). The matrix of sensitivity to absorption change (S) (which was inverted with MP) was generated considering a semi-infinite homogeneous medium^21^ and the local DS measured optical properties (also used for calculation of DPF or DSF, Secs. 2.2.1 and 2.2.2). The medium was voxelized using two layers of pillars [voxels long in z (i.e., depth)] with a lateral pitch of 1 mm (along x and y), and an axial size (along z) of 5 mm for the top-layer and 25 mm for the bottom-layer. The images reported here represent reconstructed values of the bottom-layer voxels in the x–y plane. This method for voxelizing the medium was used before for DS imaging in Refs. 21, 24.

##### Visual stimulation imaging

For the visual stimulation protocol, image reconstruction was conducted on the $\Delta \mu_{a}$ for each time-point (for each λ), resulting in an image stack of $\Delta \mu_{a}$. Then, Beer’s law was used to create image stacks of ΔO and ΔD as discussed in Sec. 2.2.2. The folding average was done across time, thus done temporally over the 11 stimulus and rest periods (Fig. 3). Two 10 s temporal windows of the image stack were selected to represent the stimulus and rest, respectively. The stimulus window ended 1 s before the end of the 15 s stimulus, and the rest window was centered in the 30 s rest period. Considering these temporal windows of image stacks, a t-test (α=0.05) was conducted for every pixel. For ΔO, the alternate hypothesis was that stimulus ΔO was greater then rest ΔO, whereas for ΔD it was that stimulus ΔD was less than rest ΔD. A significant activation Boolean spatial mask was made by only considering pixels where both ΔO significantly increased and ΔD significantly decreased during stimulus compared to rest. In addition to the boolean mask, an activation amplitude image was also created. For this, the image stacks of ΔO and ΔD were subtracted resulting in an image stack of Oxy-hemoglobin minus deoxy-hemoglobin concentration change (ΔÐ=ΔO−ΔD) [a surrogate measurement of blood-flow (BF)].^37^^,^^38^ The average ΔÐ=ΔO−ΔD was found for both the stimulus and rest 10 s windows, and the activation amplitude was taken to be the difference between the two (i.e., $\Delta {Ð}_{\mathrm{stim}}-\Delta {Ð}_{\text{rest}}=(\Delta O_{\mathrm{stim}}-\Delta O_{\text{rest}})+(\Delta D_{\text{rest}}-\Delta D_{\mathrm{stim}})$). This ΔÐ=ΔO−ΔD amplitude map was masked by the boolean mask of significant activation found via t-test to result in the activation images presented (Sec. 3.2).

##### Systemic ABP oscillations phasor imaging

The systemic ABP oscillation protocol required a different workflow to result in reconstructed images. The images sought in this case were maps of the amplitude and phase of the phasor ratio vector between deoxy-hemoglobin and oxy-hemoglobin ($\overset{˜}{D}/\overset{˜}{O}$), and the phasor ratio vector between total-hemoglobin and ABP ($\overset{˜}{T}/\overset{˜}{A}$) at 0.11 Hz. The methods in Sec. 2.2.3 output $\overset{˜}{O}/\overset{˜}{A}$ and $\overset{˜}{D}/\overset{˜}{A}$ for each measurement set in the array. Using Beer’s law,^33^ these were converted to the phasor ratio vector between absorption coefficient and ABP (${\overset{˜}{\mu }}_{a}/\overset{˜}{A}$) at each λ. From here, MP was applied to the same S as above and image reconstruction was conducted on the complex numbers representing ${\overset{˜}{\mu }}_{a}/\overset{˜}{A}$. These spatial maps of ${\overset{˜}{\mu }}_{a}/\overset{˜}{A}$ at the two λ were then converted to maps of $\overset{˜}{O}/\overset{˜}{A}$ and $\overset{˜}{D}/\overset{˜}{A}$, again using Beer’s law.^33^ Finally, maps of $\overset{˜}{D}/\overset{˜}{O}$ were created using the ratio of $\overset{˜}{O}/\overset{˜}{A}$ and $\overset{˜}{D}/\overset{˜}{A}$, and maps of $\overset{˜}{T}/\overset{˜}{A}$ using their sum. These maps of complex numbers were then smoothed using a Gaussian filter with characteristic length equal to the average array resolution^21^ to remove artifacts created by applying MP to complex numbers. The amplitude and phase of these maps of $\overset{˜}{D}/\overset{˜}{O}$ and $\overset{˜}{T}/\overset{˜}{A}$ are visualized and presented herein (Sec. 3.3).

#### 2.2.5 Data quality evaluation

To ensure that only sufficiently good-quality data were used for further analysis and image reconstruction, each data set was tested in terms of noise, coherence, signal amplitude, or voxel sensitivities. Bad sets were eliminated so they were not considered in analysis and their sensitivity region not included in S (i.e., the region under a bad set did not contain voxels used in image reconstruction). For both the visual stimulation and systemic ABP oscillations, a threshold on the noise was applied. This threshold was evaluated by first high-pass filtering to 1.7 Hz (i.e., above heart rate) to eliminate power from physiological oscillations. Then the average of the sliding windowed standard deviation (window size of 10 s) was taken as the noise amplitude (corrected for power lost at low f from the filter, assuming white noise). Channels with higher noise amplitude than 1  μM in Total-hemoglobin concentration change (ΔT) were considered bad and excluded from further analysis as noise of this amplitude would dominate over responses associated with functional of physiological cerebral hemodynamics.

For the ABP oscillations data, further quality evaluation was conducted beyond the wavelet C analysis described in Sec. 2.2.3.^34^ Any voxels with S below the $1^{\mathrm{st}}$ percentile of all S in S were ignored, as well as any measurement pairs that measured less than $0.001\;\mu M\;{\mathrm{mm}}^{-1}\;{\mathrm{Hg}}^{-1}$ in amplitude. The reasoning for the former being that voxels with small S will create large artifacts in image reconstruction (only partially addressed by Tikhonov regularization), and the reason for the latter being that one cannot claim that such a small amplitude transfer function vector was measurable considering the noise in the system. In fact, an amplitude below $0.001\;\mu M\;{\mathrm{mm}}^{-1}\;{\mathrm{Hg}}^{-1}$ would correspond to an immeasurably small oscillation in cerebral hemodynamics on the order of 0.01  μM considering a typical ABP oscillation amplitude on the order of $10\;\mathrm{mm}\;{\mathrm{Hg}}^{-1}$.

## 3 Results

### 3.1 In-Silico Investigation of Optimal Source–Detector Distances

**Fig. 4:**
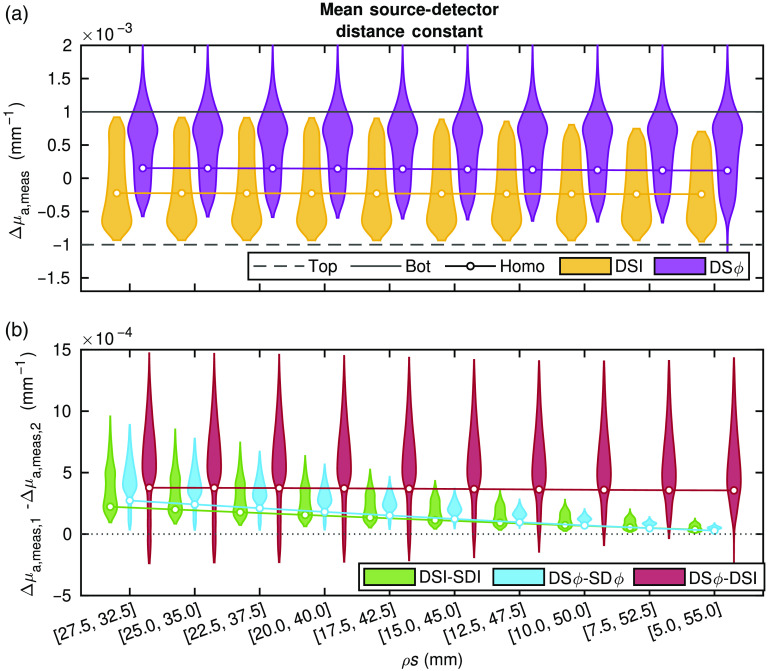
Simulated absorption coefficient change ($\Delta \mu_{a}$) obtained from various media. Specifics of the source–detector distances (ρs) including their means, maxima, and differences are shown in Table 1. About 16,807 simulations of different two-layer media were conducted (for each set of ρ), plotted as violins. Also, a representative medium with homogeneous (homo) absolute optical properties was simulated and the average measured $\Delta \mu_{a}$ (averaged over all possible layer thicknesses) recovered. In all cases, the actual $\Delta \mu_{a}$ was $-0.001\;{\mathrm{mm}}^{-1}$ in the top layer and $0.001\;{\mathrm{mm}}^{-1}$ in the bottom layer. The two ρs in a DS set were varied such that their mean ρ was constant and their difference increased. (a) Violin plots (from all 16,807 two-layer simulations) and lines with points (from example homogeneous simulation) showing the measured $\Delta \mu_{a}$ from DS Intensity (I) and DS phase (ϕ). (b) Violin plots and lines (all 16,807 two-layer and example homogeneous, respectively) showing differences between two measurement types, either DSI and long SD I, DS ϕ and long SD ϕ, or DSϕ and DS I.

**Fig. 5:**
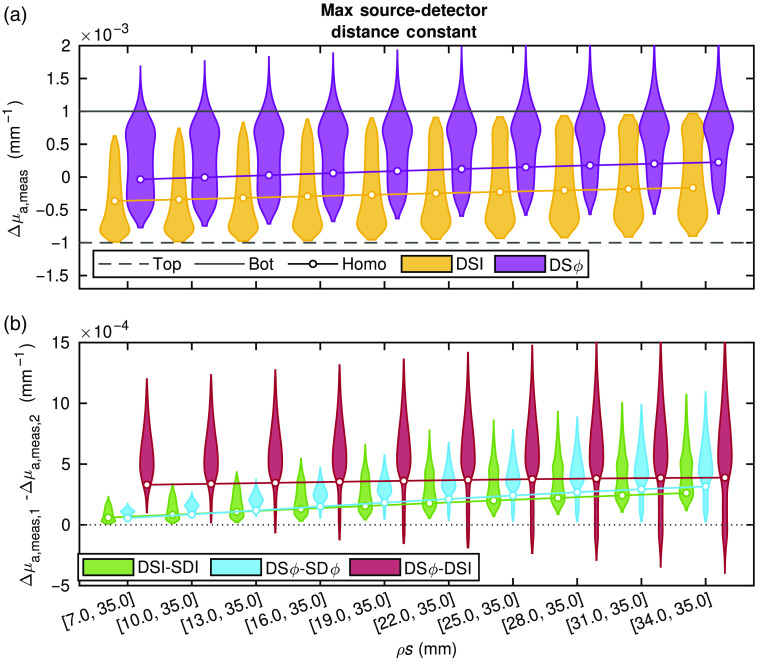
Simulated absorption coefficient change ($\Delta \mu_{a}$) obtained from various media. Specifics of the source–detector distances (ρs including their means, maxima, and differences are shown in Table 1. About 16,807 simulations of different two-layer media were conducted (for each set of ρ), plotted as violins. Also, a representative medium with homogeneous (homo) absolute optical properties was simulated and the average measured $\Delta \mu_{a}$ (averaged over all possible layer thicknesses) recovered. In all cases the actual $\Delta \mu_{a}$ was $-0.001\;{\mathrm{mm}}^{-1}$ in the top layer and $0.001\;{\mathrm{mm}}^{-1}$ in the bottom layer. The two ρs in a DS set were varied such that their maxium ρ was constant and their difference decreased. (a) Violin plots (from all 16,807 two-layer simulations) and lines with points (from example, homogeneous simulation) showing the measured $\Delta \mu_{a}$ from DS intensity (I) and DS phase (ϕ). (b) Violin plots and lines (all 16,807 two-layer and example homogeneous, respectively) showing differences between two measurement types, either DS I and long SD I, DS ϕ and long SD ϕ, or DS ϕ and DS I.

Figures 4 and 5 report the $\Delta \mu_{a}$ obtained from data computed with diffusion theory for the various conditions described in Sec. 2.1.1. The subplots in Fig. 4 show the results for a constant mean source–detector distance ($\overset{¯}{\rho }=30\;\mathrm{mm}$), and the subplots in Fig. 5 show the results for a constant maximum source–detector distance ($\rho_{\max}=35\;\mathrm{mm}$) (Sec. 2.1.1 and Table 1). The data in Figs. 4 and 5 are reported with violin plots, which show the probability density (represented by the violin thickness) corresponding to each $\Delta \mu_{a}$ value along the vertical axis. For these simulations, there are two true $\Delta \mu_{a}s$, one of the bottom- and one of the top-layer. In general, the goal is to measure a value of $\Delta \mu_{a}$ that is close to that of the bottom-layer. The reader is reminded that the homogeneous medium considered here is homogeneous in absolute optical properties but heterogeneous in $\Delta \mu_{a}$. Understanding the recovered value for this case is quite straightforward as it is a weighted average of the Ss presented previously.^11^^,^^19^^,^^20^ This simpler interpretation is the motivation for including this case in the simulations and to allow one to connect the new results to previous work.

First, in Figs. 4(a) and 5(a), which report the $\Delta \mu_{a}$ recovered from DS I or DS ϕ, one can see that in the homogeneous medium (shown by the solid line with a circle) and two-layer simulations (violin plots) there is no notable difference among the ρ sets where $\overset{¯}{\rho }$ is constant. On the other hand, when keeping $\rho_{\max}$ constant, as the two SD that comprise the DS set become closer to each other (i.e., Δρ becomes smaller), most recovered $\Delta \mu_{a}s$ become closer to that of the bottom layer. This trend is apparent in both the two-layer media (violin plots), as the mode of the $\Delta \mu_{a}$ distribution in two-layered media (violin plots) increases toward the actual bottom layer $\Delta \mu_{a}$, and in the homogeneous medium as the solid line also approaches the actual bottom layer $\Delta \mu_{a}$. From this, it is apparent that what mostly affects the sensitivity depth of DS is $\overset{¯}{\rho }$ and not $\rho_{\max}$. The mode (the visually easy part of the violin plot to identify) of the $\Delta \mu_{a}$ distribution in two-layered media (violin plots) obtained with DS ϕ is always closer to the actual bottom layer $\Delta \mu_{a}$ compared to the mode obtained with DS I and compared to the values obtained with DS ϕ or DS I in the representative homogeneous medium. It is also worth noting that the mode of the $\Delta \mu_{a}$ distribution in two-layered media obtained with DS I is always closer to the actual top layer $\Delta \mu_{a}$ compared to the $\Delta \mu_{a}$ recovered in the homogeneous medium. One can conclude that, in general (i.e., for the majority of simulations), DS ϕ is more sensitive to the bottom layer compared to DS I, and that the DS ϕ more closely retrieves the $\Delta \mu_{a}s$ that occur deeper in a two-layered medium than in a homogeneous medium.

The difference between data-types can be evaluated by examining Figs. 4(b) and 5(b). It is worth noting that here only the longer SD (i.e., SD data that feature the deepest sensitivity) is considered in the differences. In the case of DS I minus SD I (green) and DS ϕ minus SD ϕ (blue), in both sets of simulations (two-layered media and homogeneous medium), the difference between data-types is positive and moves toward zero as Δρ increases. In Fig. 4(b), where the DS sensitivity depth is about constant because of the constant $\overset{¯}{\rho }$, this is due to the increase in depth sensitivity of SD data at longer ρs. In Fig. 5(b), where the SD sensitivity depth is constant because of the constant $\rho_{\max}$, this is due to the decrease in depth sensitivity of DS data as Δρ becomes larger. It is important to note, however, that DS data (both I and ϕ) always result in a greater $\Delta \mu_{a}$ than the corresponding SD data, indicating a stronger sensitivity to the bottom layer achieved by DS versus SD data. Now focusing on DS ϕ and DS I, the difference between the associated values of $\Delta \mu_{a}$ (red) across different sets of ρs is almost constant [Figs. 4(b) and 5(b)]. This indicates that variations in neither $\overset{¯}{\rho }$ nor $\rho_{\max}$ significantly affect the relationship between the $\Delta \mu_{a}s$ measured by the two DSs types. The caveat being that Fig. 5(b) shows a slightly greater improvement in the depth sensitivity of DS ϕ compared to DS I as Δρ decreases. However, a clear result is that, as Δρ becomes smaller, the variance of the difference between $\Delta \mu_{a}s$ from pairs of data-types increases. Furthermore, there are special cases (expounded upon in Sec. 4) in which DS I achieves better sensitivity to the bottom layer compared to DS ϕ [as indicated by the portions of the violin plots below zero in Figs. 4(b) and 5(b)], but in general this is not the case.

### 3.2 In-Vivo Visual Stimulation

**Fig. 6:**
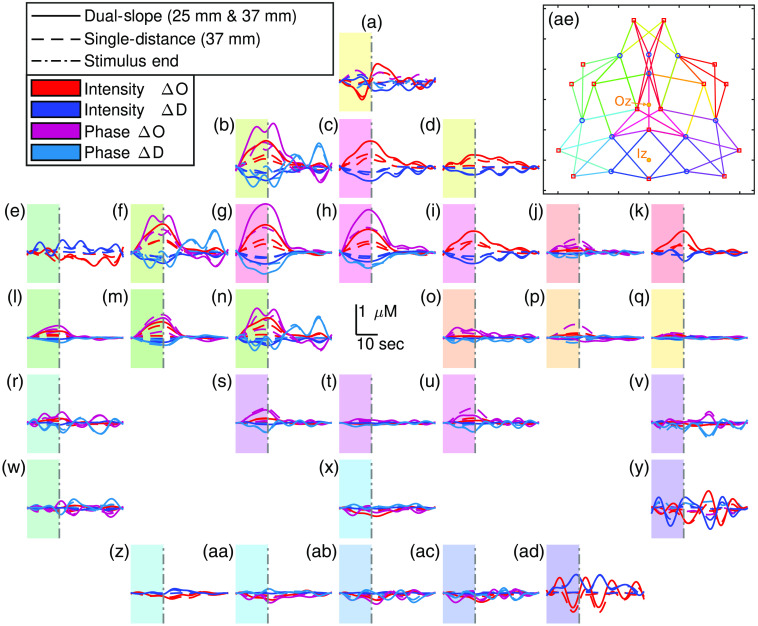
(a)–(ad) Oxy-hemoglobin concentration change (ΔO) and deoxy-hemoglobin concentration change (ΔD) of 11 repetition folding averages from an example *in-vivo* visual stimulation experiment. Locations of the insets approximately correspond to the location of each measurement set. Shaded regions indicate the activation period. A key to connect each panel to the specific DS set can be found in Fig. 2. (ae) Schematic of array from Fig. 1 indicating the approximate Iz and Oz location. Colors of the DS sets in (ae) correspond to the color of the shaded region in (a)–(ad), which also correspond to the row colors in Fig. 2. Note: traces are low-passed to 0.1 Hz.

Figure 6 shows the activation traces over the entire DS array from an example data-set of the repeated visual stimulation experiments. Each subplot represents one DS where the plot locations approximately correspond to the set location on the subject’s head. The subplots also show the data collected at the two long SD (ρ of 37 mm) pairs within each DS set. These traces are the result of low-pass filtering the data at 0.1 Hz then folding averaging across the 11 stimulation periods. The first 15 s of the trace represent the visual stimulation, whereas the following 30 s represent the rest period [Fig. 3(a)]. For Fig. 6, the characteristic functional activation response (increase in ΔO and decrease in ΔD) is localized to the upper center of the array. Additionally, for channels associated with activation, the amplitude of the functional hemodynamic response is greatest when measured with DS ϕ followed by DS I or SD ϕ then SD I (noting that SD I is the typical measurement used by CW fNIRS). The oscillations, such as the ones in Fig. 6(ad) are attributed to noise and to the cut-off frequency of the low-pass filter, showing that the 1  μM noise threshold (Sec. 2.2.5) may still allow noisy channels through the analysis.

**Fig. 7:**
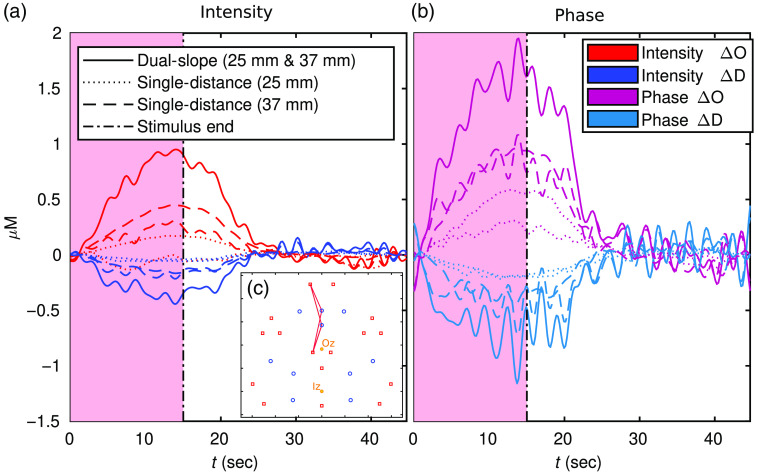
(a) and (b) Oxy-hemoglobin concentration change (ΔO) and deoxy-hemoglobin concentration change (ΔD) of an 11 repetition folding average from DS index 30 (Fig. 2) shown in Fig. 6(g). Shaded region indicates activation period and color corresponds to the color of the DS set in Fig. 1. (c) The location of the set plotted with the approximate Iz and Oz location indicated. Note: traces are low-passed to 0.5 Hz.

**Fig. 8:**
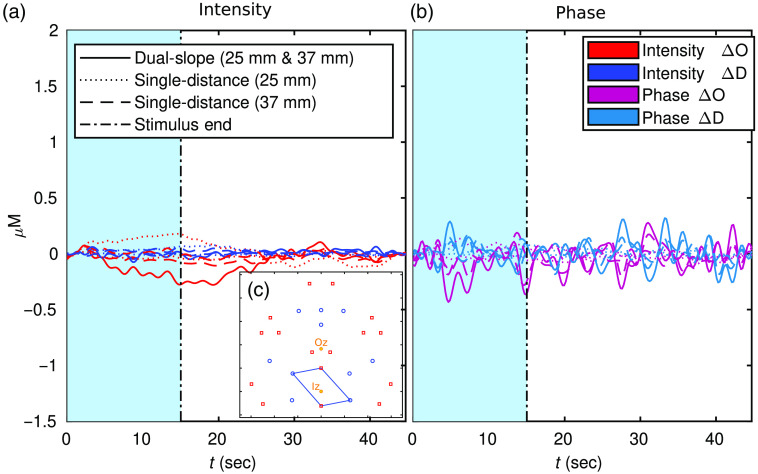
(a) and (b) Oxy-hemoglobin concentration change (ΔO) and deoxy-hemoglobin concentration change (ΔD) of an 11 repetition folding average from DS index 18 (Fig. 2) shown in Fig. 6(x). Shaded region indicates activation period and color corresponds to the color of the DS set in Fig. 1. (c) The location of the set plotted with the approximate Iz and Oz location indicated. Note: traces are low-passed to 0.5 Hz.

Figures 7 and 8, show a zoomed-in folding average of the DS data set reported in Figs. 6(g) and 6(x), respectively. These traces include all the measurements shown in Figs. 6(g) and 6(x), with the addition of the short SD within the DS set (ρ of 25 mm). In this case, the traces are low-pass filtered to 0.5 Hz then folding averaged over the 11 stimulus periods. The oscillations in Figs. 7 and 8 are due to the noise in the signal (evident in DS ϕ due to a higher noise of ϕ data overall) and the cut-off frequency of the filter (0.5 Hz).

Figure 7 shown an example of a DS set with significant activation while Fig. 8 is an example of a set without significant activation. As with the amplitude relations noted in Fig. 6, Fig. 7 also shows DS ϕ resulting in the hemodynamic response with the largest amplitude; this is followed by DS I or long SD ϕ, then long SD I or short SD ϕ, and then short SD I. It is worth noting that one short SD I data does not exhibit an activation signature, and both short SD Is measured almost no decrease in ΔD (again, SD I in the most common data-type used in typical CW fNIRS). Despite this, both short SD ϕs did measure said activation signature (including a decrease in ΔD) despite the measurements coming from the exact same optodes used to collect SD Is.

**Fig. 9:**
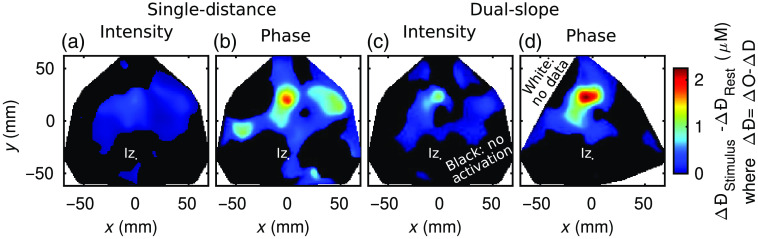
Maps of activation amplitude during the example *in-vivo* visual stimulation experiment across data-types: (a) SD intensity (I); (b) SD phase (); (c) DS I; and (d) DS ϕ. The approximate Iz location is indicated. The activation amplitude is taken to be the difference between oxy-hemoglobin minus deoxy-hemoglobin concentration change (ΔÐ=ΔO−ΔD) averages from 10 s windows during stimulus and rest. White areas show where there is no data after data quality restrictions were enforced and black areas show where activation was not significant.

The final results figure for the visual stimulation protocol is an activation image (Fig. 9). All images were reconstructed using the MP as described in Sec. 2.2.4, with 57 SD pairs or 30 DS sets used for their respective reconstructions. Following the methods in Sec. 2.2.4, the black regions of the image are areas with no significant activation, and white areas indicate locations in which no data were present (because they were either not measured or eliminated as described in Sec. 2.2.5). Significant activation was based on requiring a significant increase in ΔO and a significant decrease in ΔD using a t-test (α=0.05; Sec. 2.2.4).

The colors in the image represent the activation amplitude, based on ΔÐ=ΔO−ΔD, where the amplitude is the difference between stimulus and rest. Figure 9 shows the same relationships in activation amplitude discussed for Figs. 6 and 7, with the added caveat that SD ϕ displays a larger amplitude than DS I. Comparing the smallest amplitude to the largest, it is seen that the difference is quite stark with the activation amplitude measured with DS ϕ being about three times the one measured with SD I. Now focusing on the localization of the activation, all data-types found significant activation in the upper central area of the array (with other smaller regions possibly being false positives). This location approximately corresponds to the primary visual cortex given the array placement in relation to the Iz, a cranial landmark of the occipital pole, which is the posterior portion of the occipital lobe. Therefore, the upper portion of the array was over the occipital lobe, whereas the lower portion was over posterior neck muscles (see Fig. 1). Finally, the DS ϕ map has more white pixels, demonstrating the primary disadvantage of ϕ data, noise so that more data were eliminated by the methods described in Sec. 2.2.5.

### 3.3 In-Vivo Systemic Blood Pressure Oscillations

**Fig. 10:**
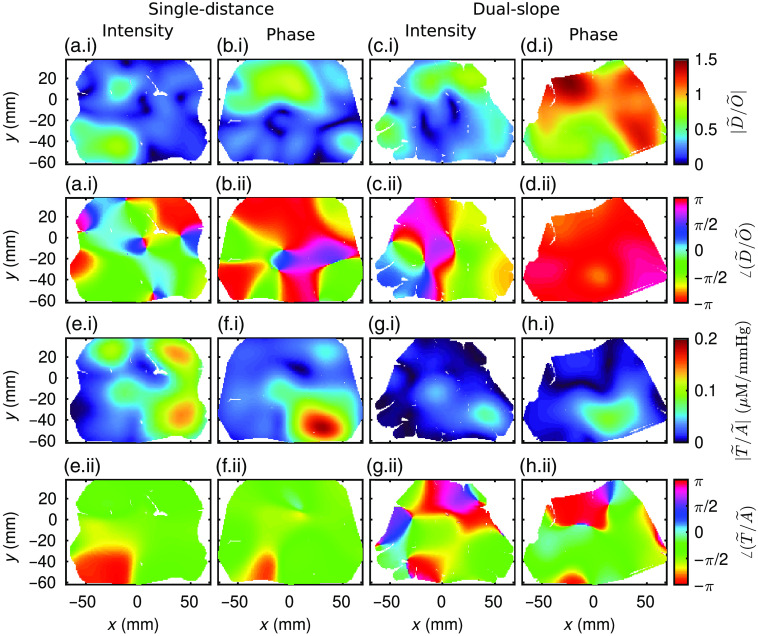
Phasor ratio vector between deoxy-hemoglobin and oxy-hemoglobin ($\overset{˜}{D}/\overset{˜}{O}$) (a)–(d) and phasor ratio vector between total-hemoglobin and ABP ($\overset{˜}{T}/\overset{˜}{A}$) (e)–(h) reconstructed maps at 0.1 Hz for SD intensity (I). (a) and (e) SD phase (ϕ). (b) and (f) DS I, and DS ϕ. (d) and (h) White areas indicate a lack of data after data quality restrictions were enforced.

Figure 10 shows the results from an example of the repeated systemic ABP oscillations experiments. Methods for image reconstruction using MP to create these images of phasor ratio vectors are described in Secs. 2.2.3 and 2.2.4. The images show either $\overset{˜}{D}/\overset{˜}{O}$ or $\overset{˜}{T}/\overset{˜}{A}$ (Figs. 10(a)–10(d) or Figs. 10(e)–10(h), respectively). Interpretation of these maps requires the simultaneous examination of the amplitude ratio ($\left|\overset{˜}{D}/\overset{˜}{O}\right|$ or $\left|\overset{˜}{T}/\overset{˜}{A}\right|$) and the phase difference ($∠(\overset{˜}{D}/\overset{˜}{O})$ or $∠(\overset{˜}{T}/\overset{˜}{A})$) of the phasors (thus, the choice of subplot lettering to include (a.i) and (a.ii), for example). This is evident in an image, such as Fig. 10(g) where the upper portion of the image has a $\left|\overset{˜}{T}/\overset{˜}{A}\right|$ close to zero, making the $∠(\overset{˜}{T}/\overset{˜}{A})$ unreliable and likely dominated by noise. With this guidance for interpretation in-mind, the two different phasor ratio pairs will now be presented in detail. All results reported here were deemed to represent hemodynamics with significant coherence (Sec. 2.2.3).^34^

$\overset{˜}{D}/\overset{˜}{O}$ represents the interplay between blood-volume (BV) and BF oscillations as described by CHS.^29^ When the vector has an angle of π rad and a magnitude of 1, the $\overset{˜}{D}$ and $\overset{˜}{O}$ are in-opposition-of-phase and have the same amplitude, thus the measured hemodynamics are dominated by BF. On the other-hand, when $\overset{˜}{D}/\overset{˜}{O}$ has an angle of 0 rad, the phasors are in-phase, and the measured hemodynamics are dominated by BV. $\overset{˜}{D}/\overset{˜}{O}$ images are shown in Figs. 10(a)–10(d). All data-types except SD I found the signature of BF-dominated hemodynamic oscillations in the upper portion of the array (corresponding to the occipital lobe) but not in the thicker tissue, including skeletal muscle, probed in the lower portion of the array (Fig. 1). In particular, DS ϕ displays BF-driven hemodynamics almost everywhere, with a higher amplitude of $\overset{˜}{D}/\overset{˜}{O}$ in the top of the image. Meanwhile, SD ϕ and DS I exhibit BF-driven hemodynamics in the upper portion of the image, but very low $\left|\overset{˜}{D}/\overset{˜}{O}\right|$ values in the lower portion, likely making the $∠(\overset{˜}{D}/\overset{˜}{O})$ in that region unreliable. Finally, the SD I image is the only one that does not display BF domination. This image [Fig. 10(a)] exhibits a low value of $\left|\overset{˜}{D}/\overset{˜}{O}\right|$ in the upper and right portions of the imaged area, again indicating unreliability of the $∠(\overset{˜}{D}/\overset{˜}{O})$ image in those regions. However, the lower left portion of the SD I image does show a larger value of $\left|\overset{˜}{D}/\overset{˜}{O}\right|$ and corresponding in-phase $∠(\overset{˜}{D}/\overset{˜}{O})$ suggesting a BV-driven hemodynamic oscillation or some combination of BF and BV contributions. In summary, DS ϕ measured a BF oscillation across the image that is strongest in the upper portion, both DS I and SD ϕ measured a BF oscillation in the upper portion of the image, and SD I measured a BV or BV mixed with BF oscillation in the lower left portion. Note again that the upper portion of the image corresponds to the occipital lobe, whereas the lower portion is likely probing the subject’s posterior neck muscles (Fig. 1).

Now, focus on the $\overset{˜}{T}/\overset{˜}{A}$ images (Fig. 10). These images relate BV oscillations (using the Total-hemoglobin phasor ($\overset{˜}{T}$) as a BV surrogate) to the $\overset{˜}{A}$. Any regions of the images with low $\left|\overset{˜}{T}/\overset{˜}{A}\right|$ are ignored since they are likely dominated by noise. From this, both DS I and ϕ in the lower right region show similar results with a larger $\left|\overset{˜}{T}/\overset{˜}{A}\right|$ amplitude and $∠(\overset{˜}{T}/\overset{˜}{A})$ being mostly in-phase or slightly negative, with DS ϕ slightly closer to 0 rad than DS I. This same sort of image is obtained with SD ϕ, only with a higher $\left|\overset{˜}{T}/\overset{˜}{A}\right|$ compared to DS I and ϕ. Note that since $\overset{˜}{A}$ is the same for all images, this means that $\overset{˜}{T}$ had a higher amplitude for SD ϕ in the lower right than DS I and ϕ. Moving to SD I, the observation is made that all regions with a low $\left|\overset{˜}{D}/\overset{˜}{O}\right|$ show a relatively large $\left|\overset{˜}{T}/\overset{˜}{A}\right|$. This means that for SD I, the entire upper portion and lower right portion of the array measured a strong $\overset{˜}{O}$ amplitude, which dwarfed $\overset{˜}{D}$, likely due to dominating BV oscillations of the arterial vascular compartment. Finally, for the SD I measurement in the lower left portion, there is a low $\left|\overset{˜}{T}/\overset{˜}{A}\right|$ meaning that the $∠(\overset{˜}{T}/\overset{˜}{A})$ value is likely unreliable. However, these results in combination with the $\overset{˜}{D}/\overset{˜}{O}$ image lead to an additional observation. In this portion of the imaged area, SD I measured $\tilde{O}$ and $\overset{˜}{D}$ both with a large amplitude and with an intermediate relative phase (i.e., between 0 rad and π/2 rad), resulting in a small $\left|\overset{˜}{T}/\overset{˜}{A}\right|$. This most probably means that SD I in this portion of the array measured a combination of BV and BF contributions to the observed hemodynamics. Finally, it is noted that SD I appears to be an outlier in that it measured hemodynamics mostly driven by BV, whereas SD ϕ, DS I, and DS ϕ all found similar results in similar regions and measured hemodynamics more strongly associated with BF.

## 4 Discussion

### 4.1 Dual-Slope Array Distance Design

The DS array used in this work is extensively described in Ref. 21, which covers the design methods to choose ρs on the basis of instrumental features and limitations. Namely, since the Imagent was considered, the array was designed to include the largest possible Δρ (to improve the signal-to-noise ratio, within the limits imposed by the available dynamic range) and $\rho_{\max}$ (to naïvely increase depth sensitivity, within the limits imposed by the requirement to collect measurable signals). Because of the goal of planar imaging and concerns of dynamic range, the array ρs where also homogenized to make all short ρs and all long ρs the same. From this, the array design featured ρs of 25 and 37 mm. Since the work herein shows the first *in-vivo* application of this DS array and DS DOI in general, a more careful examination of how these ρs affect the S depth was done via Sec. 2.1.1 and presented in Sec. 3.1 and Figs. 4–5.

Given the *in-silico* results (Sec. 3.1), it was found that the chief ρ parameter affecting S depth was $\overset{¯}{\rho }$. This may be somewhat surprising, as one may think that $\rho_{\max}$ is the important parameter (this was the naïve and incorrect assumption made in Ref. 21). Additionally, many methods and arrays in fNIRS use a very short ρ (i.e., 5 to 10 mm) for superficial hemodynamics sensing^7^^–^^10^^,^^12^ or multiple ρs including a short one,^4^^,^^16^^,^^17^ which from the perspective of these simulations would hurt S depth since $\overset{¯}{\rho }$ would be decreased. However, these methods do not utilize DS and instead typically attempt to also measure superficial hemodynamics and then remove them from long ρ data, which is not the method used here. An additional consideration of short or large ρ measurements,^4^^,^^16^^,^^17^ is the difficulty in implementing them. Measurements over a large range of ρs, where individual sources or detectors are used to collect both short and long ρs, require large dynamic range or gain switching, which increases instrumental complexity. Furthermore, short ρ measurements are prone to light leakage (i.e., detection of light that did not travel through the tissue). These considerations are not as much of a concern in DS, which utilizes two relatively long distances that can be kept within a specified range by proper deign of source–detector arrays.^21^

Looking at the DS array design (Fig. 1 and Ref. 21) with these results in mind (Sec. 3.1), one learns that the depth S of the array may have been improved by lengthening the short SD ρs. However, reducing Δρ will result in higher DS noise.^11^ Therefore, this array’s ρ set may actually achieve a good compromise between minimizing noise and maximizing S depth of DS, with a $\overset{¯}{\rho }$ of ∼31  mm, which is likely the upper end of what is achievable with the Imagent system.

One last result emerging from the *in-silico* simulations is that, on average, DS ϕ appears to achieve a better depth sensitivity than DS I in a two-layer medium, even more so than in the representative homogeneous medium. However, a previously reported two-layer case with a top-layer $\mu_{a}$ of $0.010\;{\mathrm{mm}}^{-1}$ and $\mu_{s}^{\prime }$ of $1.2\;{\mathrm{mm}}^{-1}$ and bottom-layer $\mu_{a}$ of $0.020\;{\mathrm{mm}}^{-1}$ and $\mu_{s}^{\prime }$ of $3\;{\mathrm{mm}}^{-1}$ reported that DS I exhibited deeper S than DS ϕ for a range of $z_{\text{top}}$ 4 to 10 mm.^25^ That work showed that this was consistent with a small population of *in-vivo* hemodynamics measurements in the human brain. From the simulations here, it is seen that this was a special and niche case, as confirmed by examining the DS ϕ minus DS I violin plots (red) in Figs. 4(b) and 5(b), which only show a small tail below zero (below zero meaning DS I has deeper S than DS ϕ).

To find cases that have a similar result to Ref. 25 one may look at the negative tail of the difference between DS ϕ and DS I for the ρ case of 25 and 35 mm in Fig. 5(b). This tail contains 1113 of the total 16,807 combinations of optical properties and layer-thicknesses including the specific case from Ref. 25. Furthermore, all of these cases are ones in which the top-layer $\mu_{s}^{\prime }$ is greater than the bottom-layer $\mu_{s}^{\prime }$, although these 1113 cases are not all of the instances where this scattering relationship is present (i.e., a total of 7203 cases). For $\mu_{a}$ there is no consistent relationship between the 1113 cases and the $\mu_{a}s$ can be found to take the full range of values. $z_{\text{top}}$ however does only take a certain range of values such that only cases where it is ≤8.3  mm are present in the 1113 (for all the varied values of $z_{\text{top}}$ the minimum was 5 mm and the next value above 8.3 mm was 10 mm). Therefore, here an extension of the conclusion from Ref. 25 is drawn, being that in niche cases DS I may have deeper S than DS ϕ when $\mu_{s}^{\prime }$ is greater in the top-layer for relatively thin $z_{\text{top}}$ with little influence from $\mu_{a}$.

To further investigate specific combinations of two-layer optical properties, an investigation was also done to find cases that recover the true bottom-layer $\Delta \mu_{a}$. For this investigation, only the ρs of 25 and 35 mm were considered. Qualitatively, it can be seen that most cases that recover the bottom-layer $\Delta \mu_{a}$ belong to DS ϕ (Figs. 4 and 5). Quantitatively, the number of cases out of 16807 can be counted, which recover a $\Delta \mu_{a}$ within ±10% of the true bottom-layer $\Delta \mu_{a}$. This was 1862/16807 for DS ϕ, 364/16807 for the long SD ϕ, 29/16807 for the short SD ϕ, and 9/16807 for DS I. Neither long nor short SD I ever recover values within ±10% of the true bottom-layer, and furthermore if the threshold was set at ±5% no DS I cases would meet the requirement either. To examine which combinations of optical properties result in preferential recovery of the bottom-layer, the 1862 cases in which DS ϕ reconstructed a value within ±10% of the true bottom-layer $\Delta \mu_{a}$ are considered next. None of these cases are ones in which the top-layer $\mu_{s}^{\prime }$ is greater than the bottom-layer $\mu_{s}^{\prime }$ and the top-layer $\mu_{a}$ is less than the bottom-layer $\mu_{a}$. However, cases exist in every other scenario. Considering $z_{\text{top}}$, it is noticed that the largest thickness, which meets this requirement, increases as the bottom-layer $\mu_{a}$ is less than the top-layer $\mu_{a}$. Therefore, it is concluded that the best case scenario is when the top-layer $\mu_{s}^{\prime }$ is less than the bottom-layer $\mu_{s}^{\prime }$ and the top-layer $\mu_{a}$ is greater than the bottom-layer $\mu_{a}$, with the opposite being the worst case.

Finally, it is noted that due to the negative S regions of DS and ϕ data and opposite changes simulated in the top- and bottom-layers, these simulations may artificially give a perceived advantage to DS and ϕ. This would occur by a superficial negative S of a negative $\Delta \mu_{a}$ resulting in a positive recovered $\Delta \mu_{a}$ that would be identified as coming from the bottom layer. This effect can been seen in Figs. 4(a) and 5(a) where DS ϕ can recover a $\Delta \mu_{a}>0.001\;{\mathrm{mm}}^{-1}$. To confirm that the results and conclusions in this work were not biased by this effect, additional *in-silico* simulations were done with $\Delta \mu_{a}$ only in the top- or only in the bottom-layer. These additional simulations (not presented here for conciseness) confirmed the results and conclusions discussed in this work and show that this possible bias did not affect the conclusions.

### 4.2 Functional Activation Amplitude Measured with Different Data-Types

The visual stimulation protocol based on a reversing checkerboard has been extensively studied and utilized in the field of fNIRS.^4^^,^^28^^,^^39^^–^^41^ To name a few, the protocol has been used to study cytochrome-c-oxidase (CCO) redox changes during brain activation,^39^ assess the reproducibility of fNIRS,^40^ co-register high-density DOI and fMRI,^4^ study the effect of checkerboard reversing frequency^28^ (with 8 Hz resulting in the largest amplitude of activation, thus justifying the choice made in this work), and monitor glaucoma patients.^41^ Therefore, in this work neither the protocol nor the measured hemodynamic response is the focus. Instead the visual stimulation protocol has been used as a standard protocol to test and demonstrate the DS DOI array,^21^ and compare the various data-types measurable with FD SD and DS.

Though it is not the goal of this work, a comparison of these single-subject results can be made against previously published fNIRS results, which did in-fact intend to study the cerebral hemodynamics and activation location. First, considering the spatial pattern of activation, previous work tends to present bi-lateral regions of activation.^28^^,^^40^ Compared to Fig. 9, it is noticed that only SD ϕ, and to some extent SD I, data shows this pattern, and off-center at that. The off-center nature of the image would imply array mis-alignment (i.e., the Iz not being the in the center if the array as expected), which is a possibility. The lack of bi-lateral regions in the DS images may be due to a lack of resolution or a loss of data in one region due to noise, this is particularly possible for DS ϕ. However, it should be noted that previous results are really only comparable to SD, and SD I in particular since DS is expected to give different (i.e., hopefully more brain specific) measurement results. Second, a comparison can be made against previous hemodynamic time traces.^4^^,^^28^^,^^41^ Figures 6 and 7 show various activation ΔO and ΔD amplitudes for different data-types. Again, previous work should only be compared to SD I, which showed a maximal ΔO amplitude of about 0.4  μM and a ΔD amplitude of about −0.2  μM in Fig. 7. This is almost exactly the same as the amplitudes reported in Ref. 4, about half the amplitude reported in the largest response of Ref. 28, and almost the same as the amplitude reported in Ref. 41. However, these comparisons are tenuous since different methods were used in these works. For example, Ref. 4 reports the traces of a reconstructed voxel based on a magnetic resonance imaging (MRI) informed optical property reconstruction prior and Ref. 41 utilized TD and a multi-layer reconstruction. Therefore, despite the general agreement of the amplitudes, a quantitative comparison is not truly possible. Regardless, the goal of this work does not hinge on the results exactly matching previously published works. This is mainly due to two factors. First, that only one subject is presented here since this is a technology development work rather than a study of cerebral dynamics. Second, the array used in this work is rather different than other arrays given that it is designed for DS. Even the SD results are likely not directly comparable to previous work since the arrangement is rather sparse and contains a small range of distances compared to high density DOI.^42^

Comparing the data-types, the higher amplitude response was measured by DS ϕ, followed by SD ϕ, then DS I, and SD I (Figs. 6, 7, and 9). This is consistent with the expected sensitivity depth relationships seen in Figs. 4 and 5 and presented in Refs. 11, 19, and 20. One striking result along these lines is that a given pair of optodes may measure no activation response for one data-type but a significant response for another. This was seen in Fig. 7 where a short SD I trace displayed no activation, but SD ϕ from the same pair of optodes did measure a response. Therefore, if one utilizes FD NIRS the ϕ data should not be ignored since deeper cerebral hemodynamics may be missed by only using I data. Furthermore, the results also show a further improvement achieved by DS, with DS ϕ finding the highest amplitude. This, combined with DS being insensitive to instrumental drifts and a variety of artifacts,^22^^,^^23^ leads to the recommendation that DS is explored more in fNIRS. This may be possible with existing data-sets since valid DS sets exist in many optical arrays used for fNIRS, and these DS sets can be found using the methods described in Ref. 21.

### 4.3 Spatial Mapping of Hemodynamic Oscillations

Summarizing the observations made about Fig. 10 in Sec. 3.3, SD ϕ, DS I, and DS ϕ measured similar hemodynamic phasor relationships in similar spatial regions while SD I retrieved different dynamics. Briefly, all data-types except SD I found BF dominated oscillations in the upper portion of the imaged area, corresponding to the occipital lobe (Figs. 1 and 10), and found BV oscillations in the lower right portion (mostly evident in SD ϕ and DS I), corresponding to neck muscles. It is also noted that for DS ϕ some mixture of contributions from BV and BF oscillations may have actually been measured in the lower right portion. Meanwhile, SD I found BV dominated oscillations almost across the entire image except the lower left portion, which measured a combination of BV and BF oscillations.

These results are consistent with previous work using CHS, which found that during ABP oscillations there is a transition between mainly measuring BV oscillations when probing more superficial tissue to measuring more BF oscillations when probing deeper tissue, as BF changes appear to dominate over BV changes in cerebral tissue.^35^^,^^43^ It is indeed reasonable to expect that ABP oscillations result in stronger BV oscillations in scalp tissue than in brain tissue, which is confined within the rigid skull enclosure. This is consistent with previous work considering CHS or DS^11^^,^^35^^,^^43^ and may help explain the seemingly unresolved paradoxical results showing cerebral BV changes despite the incompressibility and rigidity of the brain fluid dynamic system.^44^ These comparisons to previous works all focus on the phase and amplitude relationships of different data-types, not on the spatial dependence of the hemodynamics shown in Fig. 10. This is because, to the best of the author’s knowledge, spatial mapping of hemodynamic transfer functions measured with NIRS and CHS has not yet been presented. This makes these results possibly the most novel part of this work. For this reason it is hard to compare the spatial maps presented in Fig. 10 to previously published studies.

One way to examine the spatial mapping results is in terms of the effect of superficial hemodynamics on measurements over the whole array. Contact pressure between the imaging array and the scalp can affect the superficial hemodynamics^45^ and can create inhomogenieties across the array if contact pressure is not consistent throughout the entire array. This is particularly a concern when a measurement is dominated by superficial dynamics since the results may be significantly impacted by the way in which the optical probe is applied on the subject’s head. In these studies, no control was put in place to ensure that contact pressure did not suppress superficial hemodynamics, nor was contact pressure measured or controlled. This is one possible explanation for the inhomogeneity in measured amplitude ratio and phase difference for $\overset{˜}{D}/\overset{˜}{O}$ and $\overset{˜}{T}/\overset{˜}{A}$, especially for SD or I data-types. Future work will include exploring methods to secure the imaging array with even contact pressure that is monitored and controlled throughout the measurement.

## 5 Conclusion

This work sought to investigate and demonstrate DS DOI via three experiments. The first experiment, which was *in-silico*, examined the ρs used in a DS set and found that achieving a large mean ρ is important to maximize depth sensitivity. This first experiment justified the choice of ρs used in the DS array, which features a mean ρ of 31 mm for DS measurements considering the limitations of the Imagent. Moving from the simulations, the second and third experiments were the first *in-vivo* demonstration of DS imaging. The visual stimulation protocol successfully identified the expected activation signal in the primary visual cortex, and the DS ϕ data-type recorded the largest amplitude response, indicating its strongest sensitivity to cortical tissue. Then the experiment involving systemic ABP oscillations realized spatially resolved measurements of the phase and amplitude relationships between oxy-hemoglobin (O) and deoxy-hemoglobin (D) concentrations, and between total-hemoglobin (T) concentration and ABP. This experiment was also the first work showing spatial mapping of coherent hemodynamic oscillations that are the basis for CHS. In summary, this work reported the first *in-vivo* demonstration of DS DOI, which aims to apply the intrinsically deeply sensitive DS technique to fNIRS and CHS mapping.

## Code, Data, and Materials Availability

Supporting data and code are available at: https://github.com/DOIT-Lab/DOIT-Public/tree/master/DualSlopeImaging or upon reasonable request.
